# Effectiveness of seasonal malaria chemoprevention (SMC) treatments when SMC is implemented at scale: Case–control studies in 5 countries

**DOI:** 10.1371/journal.pmed.1003727

**Published:** 2021-09-08

**Authors:** Matthew Cairns, Serign Jawo Ceesay, Issaka Sagara, Issaka Zongo, Hamit Kessely, Kadidja Gamougam, Abdoulaye Diallo, Johnbull Sonny Ogboi, Diego Moroso, Suzanne Van Hulle, Tony Eloike, Paul Snell, Susana Scott, Corinne Merle, Kalifa Bojang, Jean Bosco Ouedraogo, Alassane Dicko, Jean-Louis Ndiaye, Paul Milligan

**Affiliations:** 1 International Statistics and Epidemiology Group, London School of Hygiene & Tropical Medicine, London, United Kingdom; 2 Medical Research Council Unit The Gambia, London School of Hygiene & Tropical Medicine, London, United Kingdom; 3 Malaria Research and Training Centre, Bamako, Mali; 4 Institut de Recherche en Sciences de la Santé, Bobo-Dioulasso, Burkina Faso; 5 Centre de Support en Santé Internationale, N’Djamena, Chad; 6 Universite Cheikh Anta Diop, Dakar, Senegal; 7 Jedima International Health Consult, Lagos, Nigeria; 8 Malaria Consortium, Kampala, Uganda; 9 Catholic Relief Services, Dakar, Senegal; 10 Faculty of Epidemiology and Population Health, London School of Hygiene & Tropical Medicine, London, United Kingdom; 11 Special Programme for Research and Training in Tropical Diseases, World Health Organization, Geneva, Switzerland; 12 University of Thies, Thies, Senegal; Mahidol Oxford Tropical Medicine Research Unit, Faculty of Tropical Medicine, Mahidol University, THAILAND

## Abstract

**Background:**

Seasonal malaria chemoprevention (SMC) has shown high protective efficacy against clinical malaria and severe malaria in a series of clinical trials. We evaluated the effectiveness of SMC treatments against clinical malaria when delivered at scale through national malaria control programmes in 2015 and 2016.

**Methods and findings:**

Case–control studies were carried out in Mali and The Gambia in 2015, and in Burkina Faso, Chad, Mali, Nigeria, and The Gambia in 2016. Children aged 3–59 months presenting at selected health facilities with microscopically confirmed clinical malaria were recruited as cases. Two controls per case were recruited concurrently (on or shortly after the day the case was detected) from the neighbourhood in which the case lived. The primary exposure was the time since the most recent course of SMC treatment, determined from SMC recipient cards, caregiver recall, and administrative records. Conditional logistic regression was used to estimate the odds ratio (OR) associated with receipt of SMC within the previous 28 days, and SMC 29 to 42 days ago, compared with no SMC in the past 42 days. These ORs, which are equivalent to incidence rate ratios, were used to calculate the percentage reduction in clinical malaria incidence in the corresponding time periods. Results from individual countries were pooled in a random-effects meta-analysis. In total, 2,126 cases and 4,252 controls were included in the analysis. Across the 7 studies, the mean age ranged from 1.7 to 2.4 years and from 2.1 to 2.8 years among controls and cases, respectively; 42.2%–50.9% and 38.9%–46.9% of controls and cases, respectively, were male. In all 7 individual case–control studies, a high degree of personal protection from SMC against clinical malaria was observed, ranging from 73% in Mali in 2016 to 98% in Mali in 2015. The overall OR for SMC within 28 days was 0.12 (95% CI: 0.06, 0.21; *p <* 0.001), indicating a protective effectiveness of 88% (95% CI: 79%, 94%). Effectiveness against clinical malaria for SMC 29–42 days ago was 61% (95% CI: 47%, 72%). Similar results were obtained when the analysis was restricted to cases with parasite density in excess of 5,000 parasites per microlitre: Protective effectiveness 90% (95% CI: 79%, 96%; P<0.001), and 59% (95% CI: 34%, 74%; P<0.001) for SMC 0–28 days and 29–42 days ago, respectively. Potential limitations include the possibility of residual confounding due to an association between exposure to malaria and access to SMC, or differences in access to SMC between patients attending a clinic and community controls; however, neighbourhood matching of cases and controls, and covariate adjustment, attempted to control for these aspects, and the observed decline in protection over time, consistent with expected trends, argues against a major bias from these sources.

**Conclusions:**

SMC administered as part of routine national malaria control activities provided a very high level of personal protection against clinical malaria over 28 days post-treatment, similar to the efficacy observed in clinical trials. The case–control design used in this study can be used at intervals to ensure SMC treatments remain effective.

## Abstract 2

Using case-control studies, Matthew Cairns and colleagues investigate the effectiveness of seasonal malaria chemoprevention against clinical malaria in children in Burkina Faso, Chad, Mali, Nigeria and The Gambia.

## Author summary

**Why was this study done?:**

- Seasonal malaria chemoprevention (SMC) consists of monthly administration of 2 antimalarial drugs, sulphadoxine–pyrimethamine and amodiaquine, to children 3–59 months of age during the peak months of malaria transmission.
- SMC provided a high level of protection against clinical malaria in clinical trials, and has been recommended by the World Health Organization since 2012 for areas of the Sahel and sub-Sahel regions of Africa with seasonal transmission. This study was done to determine whether the efficacy observed in the trials translated into effectiveness when SMC was deployed at scale.

**What did the researchers do and find?:**

- The researchers carried out case–control studies in Burkina Faso, Chad, Mali, Nigeria, and The Gambia to estimate the protective effectiveness of SMC treatments against clinical malaria.
- Cases were children with confirmed malaria at a health facility. For each case, 2 controls were chosen from the neighbourhood where the case lived. Dates of SMC treatments were determined from SMC record cards and by asking caregivers. The effectiveness of monthly SMC treatment in preventing clinical malaria was estimated by comparison of when cases and controls had most recently received SMC.
- In all 7 case–control studies (2 in 2015 and 5 in 2016), SMC provided a high level of protection against clinical malaria. Protection was highest in the first 4 weeks after treatment. Pooling estimates across the 7 studies, clinical malaria incidence during the first 4 weeks after SMC treatment was reduced on average by 88%. Protection from SMC was lower in the period 5–6 weeks post-administration.

**What do these findings mean?:**

- SMC was highly effective in preventing clinical malaria when delivered at scale through national malaria control programmes, with protection comparable to that observed during clinical trials. This case–control design can be used by national malaria control programmes at intervals to confirm that SMC remains effective.

## Introduction

Seasonal malaria chemoprevention (SMC) is a relatively new tool for the prevention of malaria in areas with seasonal transmission. SMC consists of monthly administration of a full therapeutic course of the antimalarials sulphadoxine–pyrimethamine (SP) and amodiaquine (AQ) during the peak malaria season, and has been shown to provide a high level of protection against malaria in a series of clinical trials [1]. Since being recommended by the World Health Organization in 2012 [2], SMC has been introduced as part of national malaria control strategies in countries of the Sahel and sub-Sahel regions of West Africa, and reached 22 million children in 13 countries in 2019 [3].

With SMC deployed at scale, there is a need to confirm if the high level of protection against clinical malaria observed in clinical trials is replicated in a programmatic field setting, as a number of factors (such as drug quality, poor administration, poor adherence to the 3-day regimen, and the presence of illnesses that affect drug absorption) could limit treatment efficacy in practice if SMC delivery is not well supervised. There is also an ongoing need to monitor SMC effectiveness, which could decline over time if parasites become more resistant to SP or AQ, or both, in regions where SMC is being used. Prior to widespread implementation of SMC, the frequencies of molecular markers of resistance to both SP and AQ were low in countries that now have SMC programmes [4], but this situation could change. Since the relationship between marker prevalence and the protective efficacy (PE) of chemoprevention is not straightforward [5,6], monitoring the prevalence of these molecular markers alone will not be sufficient to understand the potential impact of changing drug resistance patterns on SMC efficacy, and epidemiological studies of effects on malaria incidence are needed. Other studies have measured the impact of SMC on prevalence of asymptomatic *Plasmodium falciparum* infection and the prevalence of fever [7–9], but the effectiveness of SMC in protecting children against clinical malaria requiring treatment at a health facility is the key knowledge gap, since this measure would be more easily interpreted.

Case–control studies are logistically less complex than cohort studies and are commonly used to estimate the protective effectiveness of vaccines post-implementation [10]. Case–control studies have also been used to evaluate other preventive interventions for malaria, such as treated bednets [11,12]. As usually designed, with controls recruited concurrently as cases occur, case–control studies provide an estimate of the incidence rate ratio for the exposure [13,14], and hence the protective effectiveness, i.e., the percentage reduction in disease incidence associated with the exposure, calculated as 100 × (1 − rate ratio). However, case–control studies need to be planned carefully in order to ensure that receipt of the intervention (the exposure) is accurately measured, and that the controls represent the population that produced the cases.

The ACCESS-SMC project evaluated the impact and effectiveness of SMC delivered in 7 countries of the sub-Sahel region of Africa during 2015 and 2016 [4]. Although delivery methods varied between countries (some countries delivered SMC door-to-door, and others at fixed points in the community), procedures when a recipient child was contacted were standardised, and the same regimen (a single treatment with SP plus AQ daily for 3 days) was used. In this paper, we report the results of case–control studies undertaken in 5 of these countries—Burkina Faso, Chad, Mali, Nigeria, and The Gambia—to estimate the protective effectiveness of SMC against clinical malaria across the region where it has been deployed.

## Methods

**Fig 1:**
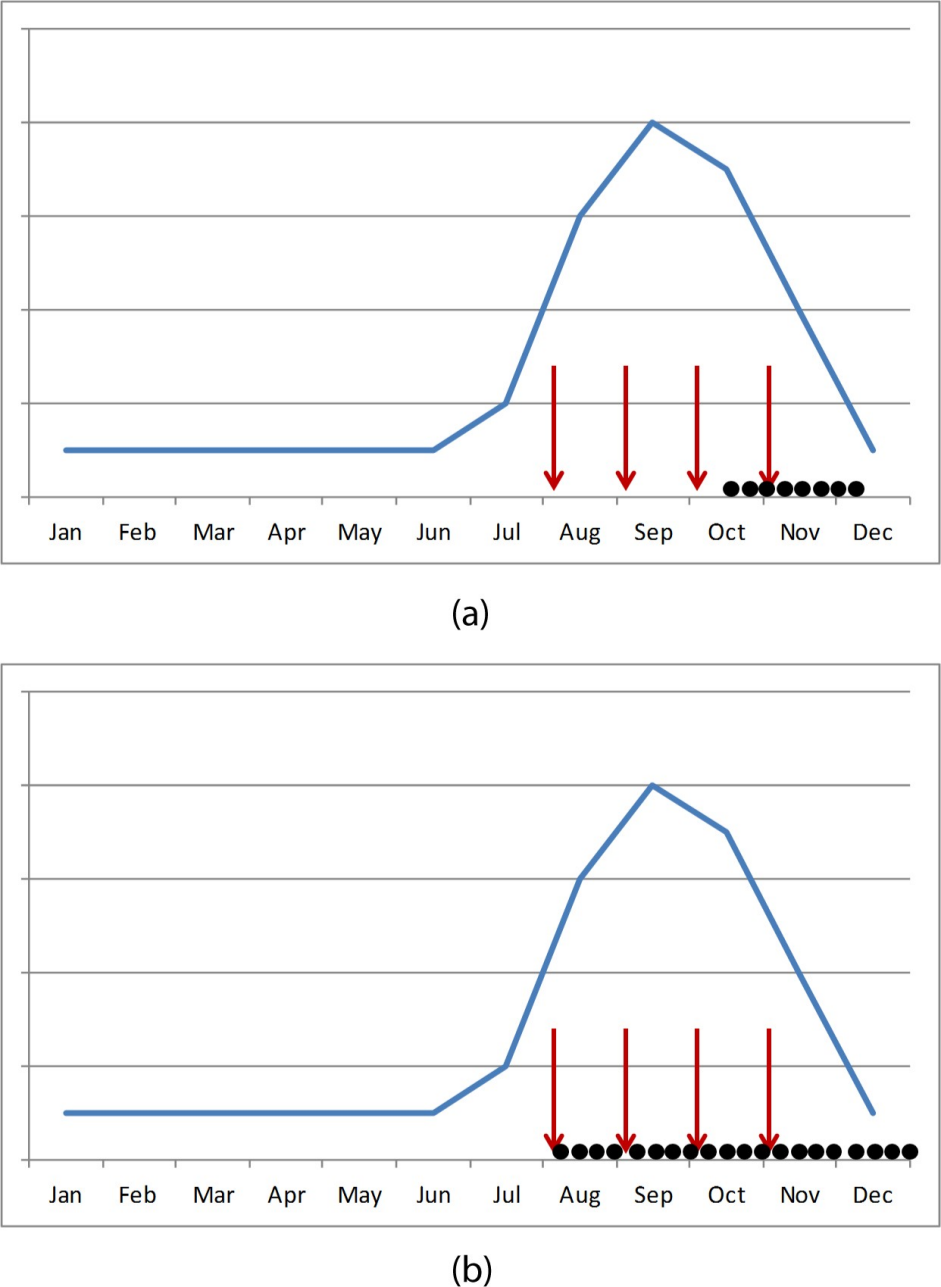
Schematic of recruitment of cases and controls in 2015 and 2016. (a) 2015; (b) 2016. This schematic of recruitment shows the timing of seasonal malaria chemoprevention (SMC) cycles (red arrows) and the recruitment period of the case–control study (black dots) in relation to the typical seasonal peak in malaria cases (hypothetical seasonality pattern shown by the blue line). In 2015, cases and corresponding controls were recruited in the late rainy season and early dry season in Mali and The Gambia. In 2016, cases and controls were recruited uniformly across the entire malaria transmission season in Burkina Faso, Chad, Mali, Nigeria, and The Gambia, from the time of the first SMC cycle until 8 weeks after the final cycle.

The outcome of interest in this study was clinical malaria, i.e., children seeking care for fever due to *P*. *falciparum* infection confirmed by blood smear. In 2015, case–control studies were conducted in Mali and The Gambia during the late rainy season and early dry season (Fig 1A), from October to December 2015 in Mali, and December 2015 to January 2016 in The Gambia. In Mali, cases were recruited at Diema and Sanso health centres, and in The Gambia, at Basse, Koina, Fatoto, Sabi, Gambissara, and Jahali health centres and Bansang Hospital, which serves the surrounding rural area.

In 2016, cases were recruited in 5 countries (Burkina Faso, Chad, Mali, Nigeria, and The Gambia) over the entire transmission season (i.e., starting from the time of the first SMC cycle) and were recruited until approximately 8 weeks after the final SMC cycle (Fig 1B), to allow protection to be estimated beyond the first month after treatment. Specific dates were as follows: Burkina Faso, August 2016 to January 2017; Chad, August 2016 to March 2017; Mali, August 2016 to January 2017; Nigeria, August 2016 to February 2017; The Gambia, September to December 2016. To meet the overall target sample size and to ensure that cases were recruited uniformly during the transmission period, and with respect to the timing of monthly SMC cycles, study teams recruited an approximate quota of cases each week (S1 Methods). In Mali and The Gambia, the same health centres were used in 2016 as in 2015, except that Bansang Hospital was not used. Cases were recruited in 2 health centres in Burkina Faso (Zitenga and Koupela), 4 health centres in Chad (1 in Koundoul and 3 in N’Djamena), and in the outpatient department of general hospitals in Nigeria (Gwadabawa and Wurno in Sokoto State; Kaura Namoda and Tsafe in Zamfara State). Apart from the 3 health centres in N’Djamena, which served a partly urban area, all clinics included in the study served a rural area.

### Recruitment of clinical malaria cases

Children aged 3–59 months presenting at health facilities with fever (axillary temperature ≥ 37.5°C or a history of fever in the last 48 hours) who did not have respiratory infection or other obvious cause of the fever were tested for the presence of malaria infection using microscopy. Microscopic examination of blood smears was used to reduce the detection of false positives that is common with rapid diagnostic tests [15]. After administering treatment, children with confirmed clinical malaria who were accompanied by a parent or legal guardian able to provide consent were invited to participate. Signed consent was sought from the caregiver after explaining the aims and procedures of the study, and the child enrolled as a case. A field worker visited the child’s home as soon as possible thereafter (usually later the same day or the next day). At the home visit, the dates of SMC treatments the case had received were determined by inspecting the child’s SMC card, if available, and by asking the caregiver to recall when the child was last treated. If there was a disagreement between the caregiver’s recollection and the card record, interviewers attempted to verify the treatment date by checking against the known dates of SMC delivery in the village and by asking other family members. Information was also collected to allow adjustment for the following potential confounding factors: age, sex, whether the child slept under a bednet the previous night, the type of net (determined by inspecting the sleeping space and the net), and caregiver’s education and socioeconomic status (SES, calculated separately for each study using principal component analysis of durable assets and amenities of the household). Information was also collected on the number of children aged 3–59 months in the household and, in 2016 only, prior antimalarial treatment (other than SMC) in the past 3 weeks, although these factors were found not to be important confounders and were not adjusted for.

### Recruitment of controls

Controls were selected from the same neighbourhood as the case (i.e., matched on neighbourhood) in order to control for potential confounding by level of exposure to malaria transmission and access to healthcare. After interviewing the case, compounds in the neighbourhood of the case were visited to recruit 2 controls from separate households. Households were visited starting at least 3 compounds away from the home of the malaria case. If there was more than 1 eligible child in a household, a Kish grid was used to select one [16]. All children aged 3–59 months were eligible as controls, including unwell children who might have had malaria (such children were then either treated or referred to health facilities for treatment). Interviewers were trained to record each household that was approached and each child invited to participate using a specific form, in order to confirm participation rates among potential controls. This was done in both years, on paper forms in 2015 (separate from the study forms on a tablet personal computer [PC]) and directly on tablet PCs in 2016. Details about SMC treatments and potential confounders for controls were collected in the same way as for cases. Interviews were timed to check that a similar length of time was spent collecting information from cases and controls.

### Primary exposure

**Fig 2:**
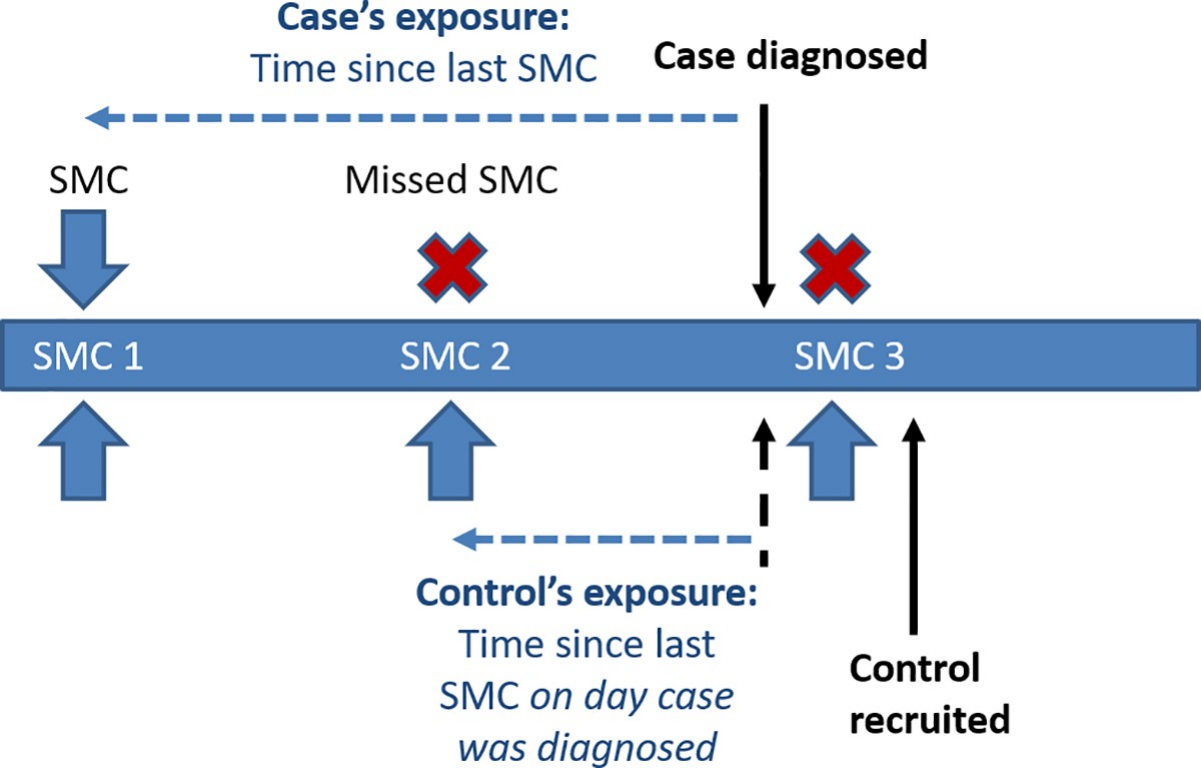
Calculation of exposure in controls with respect to the date that the case was diagnosed. For cases, the primary exposure (time since the most recent seasonal malaria chemoprevention [SMC] course) was defined on the basis of the most recent SMC cycle received at the time of diagnosis. For controls, the primary exposure was defined on the basis of the most recent SMC received at the time the case was diagnosed. On most occasions, this was straightforward, as for both case and control, the most recent SMC cycle was some time ago, and defining the exposure relied only on accurate recording of dates. However, in a few instances, the case was recruited just before a new SMC cycle was delivered, and the control was not recruited until just afterwards. The figure shows the example of a case being recruited just before SMC 3, and a control being recruited just after SMC 3. Because the exposure for the control is defined based on the most recent SMC received at the time the case is diagnosed, in this example the control’s exposure is based on SMC 2, rather than SMC 3. This avoids the possible bias of some controls appearing to have received SMC much more recently than cases (which would inflate the apparent benefit of SMC) as an artefact of the slight delay in the recruitment of controls.

The primary exposure was defined for cases and controls as the time since the first daily dose of the most recent SMC treatment, up to the time the case was diagnosed, i.e., ignoring any SMC treatment courses that were administered between recruitment of the case and recruitment of the control (Fig 2). This exposure was categorised as SMC within the previous 28 days, SMC 29–42 days ago, and no SMC within the past 42 days; these groupings were chosen because the PE from SMC against clinical malaria has been shown to be very high in the initial 4 weeks after administration, with protection then waning over the period between 4 and 6 weeks [17].

### Laboratory methods and quality control

Thick blood films were stained with Giemsa and air-dried, with 100 high-power fields read before declaring a slide negative. Parasite densities were estimated by counting against 200 white blood cells (WBCs) and assuming 8,000 WBCs per microlitre. In Nigeria, parasite density was categorised, and the exact density was not recorded. All slides were double read, with discrepancies on result or density resolved either by a third reader or, in Burkina Faso, with the expert reader rereading their slide.

### Monitoring of study activities and data management

Monitors from London School of Hygiene & Tropical Medicine and from Universite Cheikh Anta Diop in Dakar, Senegal, visited each site to check that the study protocol was being followed. In both years, data were collected on tablet PCs (Google Nexus) using the iFormBuilder platform, uploaded to a server, and imported to a Microsoft Access database, except in Nigeria (where data were collected using paper forms and then double entered into an Access database; for quality control a sample of forms were scanned and checked against the dataset). Date and other data consistency checks were carried out on the central database, with queries resolved by the teams in each centre.

### Statistical methods

All analyses were conducted in Stata 15 (StataCorp, College Station, Texas).

#### Protective effectiveness of SMC

The SMC status of cases and controls within matched sets was examined by tabulation. Only case–control sets where the case and at least 1 control were discordant for exposure contribute to the matched analysis. The timing of the most recent SMC course for cases and controls was therefore plotted within discordant case–control sets: If cases tended to have received SMC longer ago than controls, this indicates that SMC is protective. Conditional logistic regression was then used to estimate crude and adjusted (for age, sex, long-lasting insecticidal net [LLIN] use, education, and SES) odds ratios (ORs) for SMC 0–28 days and 29–42 days ago, relative to children with no SMC within the past 42 days (reference group). All models were adjusted for all of the above confounders (i.e., no variable selection), with the exception of the Mali 2015 and The Gambia 2015 studies, in which child’s sex and SES, respectively, were not captured for all children, so these were not included in the adjusted model.

As described above, due to the concurrent recruitment of controls, the OR can be interpreted as the incidence rate ratio. The protective effectiveness of SMC in the given time periods against clinical malaria was calculated as 1 − OR, expressed as a percentage. Although we did not expect the effectiveness of SMC to vary according to the length of the malaria transmission season, the study centres in Mali were in parts of the country with markedly different malaria epidemiology (Diema in the Sahelian region, 200 km north of Bamako; Sanso is approximately 400 km south of Diema), so we prespecified an analysis examining evidence for effect modification (interaction) of SMC effectiveness by study area, using the likelihood ratio test (LRT) to compare models with and without an interaction term between the SMC variable and site. Other countries were treated as a single centre. A multivariable regression model was also used to estimate the protective effectiveness of LLIN use the night before the case or control was interviewed.

#### Meta-analysis

To obtain a summary estimate of SMC protection between 0 and 28 days, and between 29 and 42 days, a random-effects meta-analysis was conducted using the log OR and its standard error from each of the 7 studies. The contribution of each study to the overall estimate and evidence for heterogeneity in the OR was estimated.

#### High-parasite-density malaria

As a secondary analysis, to increase the probability that the observed illness is attributable to *P*. *falciparum* infection (rather than another cause, with coincidental parasitaemia), the analysis described above was repeated restricted to cases with parasite density greater than 5,000 per microlitre, and their corresponding controls. This analysis excluded Nigeria, 2016, as the exact density was not available. As for children with parasitaemia of any density, results were combined in a random-effects meta-analysis as described above.

All analyses were planned before the final database was locked for analyses. However, in response to comments from the statistical peer reviewer, we subsequently carried out a fixed-effects meta-analysis to compare with the random-effects model, and calculated the prediction interval for the random-effects meta-analysis. This study is reported as per the Strengthening the Reporting of Observational Studies in Epidemiology (STROBE) guideline (S1 STROBE Checklist).

### Ethics

The protocol was approved by the London School of Hygiene & Tropical Medicine Research Ethics Committee (No. 9944, 11 August 2015) and by ethics committees in each participating country. Signed consent was obtained from caregivers for participation in the case–control studies after explaining the study aims and procedures using a standard script.

## Results

### Characteristics of cases and controls

Across the 7 studies in the 5 countries (2 in 2015 and 5 in 2016), there were generally more females than males among both controls and cases (42.2%–50.9% and 38.9%–46.9% of controls and cases, respectively, were male). Mean age was slightly lower among the controls compared to the cases (mean age for controls ranged from 1.7 years to 2.4 years, and mean age for cases from 2.1 to 2.8 years) (Table 1). LLIN use among cases and controls the night before the interview was high in most studies, ranging from 84.6% in Nigeria, 2016, to 97.5% in Burkina Faso, 2016. The exception was Chad, 2016, in which use of LLIN was only 38.6%. Mothers/caregivers of cases generally had a slightly lower education level than mothers/caregivers of controls. The distribution of education status and SES categories among the cases and controls is also shown in Table 1. Refusals to participate among controls were not systematically documented on tablet PCs in 2015, but were reported to be very rare. In 2016, there were no refusals in Burkina Faso and Nigeria, 1 each in Chad and Mali, and 5 in The Gambia.

**Table 1:** Percentage of clinical malaria cases and controls with the primary exposure and other risk factors.

		Mali, 2015		The Gambia, 2015		Burkina Faso, 2016		Chad, 2016		Mali, 2016		Nigeria, 2016		The Gambia, 2016	
		Controls	Cases	Controls	Cases	Controls	Cases	Controls	Cases	Controls	Cases	Controls	Cases	Controls	Cases
		N=504, %	N=252, %	N= 452, %	N = 226, %	N=918, %	N=459, %	N=398, %	N=199, %	N=682, %	N=341, %	N=802, %	N=401, %	N=496, %	N=248, %
Last SMC	< 28 days	46.6	6.8	12.1	8.1	72.5	59.5	20.1	12.1	36.2	20.1	46.2	36.7	79.4	57.3
	29-42 days	9.1	9.1	16.4	9.0	20.6	25.1	7.3	5.0	11.5	10.9	13.7	12.5	12.7	14.1
	43+ days	44.4	84.1	71.5	82.8	6.9	15.5	72.6	82.9	52.3	69.0	40.1	50.9	7.9	28.6
	n missing	96	32	6	5	0	0	0	0	3	2	29	0	0	0
Sex	Male	-	-	50.9	46.9	47.0	44.3	42.2	41.2	48.4	44.2	45.4	38.9	44.7	39.9
	n missing	-	-	5	2	17	1	0	0	0	2	5	0	2	0
Age	0	15.1	4.0	7.1	2.7	5.1	5.7	16.1	13.6	15.7	3.5	20.3	10.2	12.3	2.8
(years)	1	15.3	11.9	29.2	17.7	20.2	18.5	26.4	21.6	19.6	9.7	24.9	24.9	20.6	13.3
	2	21.5	20.6	25.4	27.9	29.0	29.0	33.4	26.6	19.8	25.2	25.4	27.2	25.2	28.2
	3	26.4	30.6	23.6	31.9	22.7	22.4	16.3	16.6	20.1	27.6	18.7	19.7	20.4	27.8
	4	21.7	32.9	14.7	19.9	23.1	24.4	7.8	21.6	24.8	34.0	10.6	18.0	21.6	27.8
	n missing	1	0	2	0	0	0	0	0	0	0	0	0	0	0
LLIN last	yes	96.8	96.8	86.4	75.3	97.5	93.2	38.6	43.7	96.9	97.3	84.6	63.2	85.5	83.9
Night	n missing	2	1	3	3	9	1	2	0	9	6	75	61	1	2
SES	1) highest	19.9	24.3	19.9	23.7	20.0	7.7	12.6	8.5	19.9	21.4	20.8	21.4	20.0	35.5
	2) high	20.1	20.7	19.9	20.2	20.0	18.9	24.6	15.1	19.9	20.5	20.1	19.2	20.0	20.6
	3) average	19.9	18.3	20.2	21.4	20.0	35.1	20.6	25.1	19.9	20.2	19.3	10.2	20.0	18.5
	4) low	20.1	20.3	19.9	19.1	20.0	24.3	22.1	23.6	19.9	19.1	20.4	18.2	20.0	9.3
	5) lowest	20.1	16.3	20.2	15.6	20.0	14.0	20.1	27.6	20.1	17.9	19.3	30.9	20.2	16.1
	n missing	1	1	109	53	0	0	0	0	0	0	0	0	0	0
Education*	0) Least	67.7	69.0	12.2	14.8	73.7	83.8	7.3	10.6	71.3	68.3	67.0	74.6	81.0	83.9
	1)	10.5	9.5	72.4	71.3	14.3	12.0	32.9	41.2	8.4	7.3	20.6	13.5	18.8	16.1
	2)	10.5	14.7	15.4	13.9	12.0	4.2	32.9	22.6	10.4	13.5	12.5	12.0	-	-
	3)	11.3	6.7	-	-	-	-	26.9	25.6	10.0	10.9	-	-	-	-
	n missing	0	0	2	3	18	2	0	0	0	0	0	0	1	0

Dashes indicate where information was not collected (for Sex in Mali, 2015), or where the category was not used (for Education, which was defined as follows).

*Education categories in 2015: **Mali** 0) None, 1) Koranic, 2) Primary, 3) Secondary/Higher; **The Gambia**: 0) None 1) Koranic 2) Primary/Secondary/Higher. Education categories in 2016: **BF**: 0) none, 1) primary, 2) Secondary/Higher; **Chad** and **Mali** 0) None, 1) Koranic, 2) Primary, 3) Secondary/Higher; **Nigeria**: 0) None/Koranic, 1) Primary, 2)Secondary/College; **The Gambia**: 0) None/Koranic, 1) Primary/Higher.

In all studies, the majority of controls were recruited within 2 days of the case attending the health facility. In 5 studies, all controls were recruited within 7 days. In Burkina Faso and Chad, in 2016, some controls were recruited between 7 and 30 days after the case, but the recording and analysis focused on the status of the controls at the time the case attended the health facility (Table A in S1 Tables). Availability of the SMC card for inspection at the home visit among the controls ranged from 34.0% in Mali, 2016, to 99.4% in Burkina Faso, 2016 (Table B in S1 Tables). Availability of the card was generally lower among cases (as would be expected, as children who have not received SMC would not have a recipient card).

Parasite density among the clinical malaria cases recruited for the study was generally high: The percentage of cases with parasite density greater than 5,000 parasites per microlitre ranged from 65.8% in Chad, 2016, to 83.3% in Mali, 2016 (Table C in S1 Tables). Geometric mean parasite density per microlitre ranged from 9,475 in the Gambia, 2016, to 24,191 in Mali, 2016. According to caregiver reports, all 3 daily doses of SMC were administered to a very high percentage of children in all of the studies (Table D in S1 Tables). Among controls who had received SMC, the percentage reported to have successfully swallowed all 3 daily doses ranged from 61.8% in The Gambia, 2015, to 97.1% in Burkina Faso, 2016. Caregiver-reported spitting or vomiting of at least 1 of the daily doses was variable (ranging from 2.4% in Burkina Faso, 2016, to 34.6% in The Gambia, 2015). Very few doses were missed because the child refused to take the medication (0.11% in Burkina Faso, 2016, to 5.8% in Nigeria, 2016).

The most common discrepancy between cases and controls in the primary exposure (time since SMC) was the case having not received SMC in the past 28 days, while at least 1 of the controls had received SMC in this period (Table E in S1 Tables). Cases generally had SMC longer ago than controls (Fig A in S1 Fig). Both these findings are consistent with a protective effect of SMC against clinical malaria.

### Protective effectiveness of SMC against clinical malaria by country

In 2015, 252 cases and 504 controls were recruited in Mali; 111 case–control sets were discordant for recent SMC and contributed to the analysis (Table E in S1 Tables). There were very few instances where a case, but neither control, had received SMC within the previous 28 days, leading to a very high estimate of protection from SMC in the first 28 days. There were missing data on the most recent SMC for 96 controls and 32 cases, arising from imprecise recording or recall of dates in Mali in 2015. After adjustment for age, LLIN use, SES, and education, the adjusted OR for SMC within the previous 28 days was 0.02 (95% CI: 0.01, 0.06), indicating protective effectiveness against clinical malaria of 98% (95% CI: 94%, 99.5%) (Table 2). There was no evidence of protection beyond this period, nor of heterogeneity between Diema and Sanso districts (LRT *p =* 0.78). In 2015, 226 case–control sets were recruited in The Gambia, 51 of which were discordant for SMC (Table E in S1 Tables). After adjustment for sex, age, LLIN use, and education, the adjusted OR for SMC 0–28 days ago was 0.15 (95% CI: 0.04, 0.56) and for SMC 29–42 days ago was 0.23 (95% CI: 0.10, 0.53), indicating a protective effectiveness of 85.0% (95% CI: 43.1%, 96.0%) and 77.2% (95% CI: 47.3%, 90.2%), respectively, in these 2 periods (Table 2).

**Table 2:** Association of SMC with clinical malaria in individual studies.

Study	SMC	Crude OR	95% CI	Adjusted OR	95% CI	*p-*Value	PE	95% CI
**Mali, 2015**	Within previous 28 days	0.022	0.007, 0.070	0.017	0.005, 0.059	<0.001	98.3	94.1, 99.5
	29–42 days ago	0.473	0.171, 1.308	0.482	0.162, 1.432	0.189	51.8	−43.2, 83.8
	43+ days ago	—	—	—	—	—	—	—
**The Gambia, 2015**	Within previous 28 days	0.131	0.035, 0.487	0.150	0.040, 0.569	0.005	85.0	43.1, 96.0
	29–42 days ago	0.207	0.092, 0.467	0.228	0.098, 0.527	0.001	77.2	47.3, 90.2
	43+ days ago	—	—	—	—	—	—	—
**Burkina Faso, 2016**	Within previous 28 days	0.047	0.020, 0.113	0.066	0.026, 0.169	<0.001	93.4	83.1, 97.4
	29–42 days ago	0.310	0.129, 0.743	0.428	0.160, 1.144	0.091	57.2	−14.4, 84.0
	43+ days ago	—	—	—	—	—	—	—
**Chad, 2016**	Within previous 28 days	0.317	0.163, 0.618	0.222	0.105, 0.469	<0.001	77.8	53.1, 89.5
	29–42 days ago	0.442	0.169, 1.161	0.434	0.150, 1.256	0.124	56.6	−25.6, 85.0
	43+ days ago	—	—	—	—	—	—	—
**Mali, 2016**	Within previous 28 days	0.294	0.201, 0.431	0.271	0.177, 0.415	<0.001	72.9	58.5, 82.3
	29–42 days ago	0.637	0.389, 1.042	0.537	0.314, 0.918	0.023	46.3	8.2, 68.6
	43+ days ago	—	—	—	—	—	—	—
**Nigeria, 2016**	Within previous 28 days	0.218	0.132, 0.360	0.169	0.092, 0.309	<0.001	83.1	69.1, 90.8
	29–42 days ago	0.410	0.198, 0.848	0.363	0.151, 0.873	0.024	63.7	12.7, 84.9
	43+ days ago	—	—	—	—	—	—	—
**The Gambia, 2016**	Within previous 28 days	0.081	0.041, 0.159	0.081	0.039, 0.170	<0.001	91.9	83.0, 96.1
	29–42 days ago	0.267	0.121, 0.588	0.221	0.092, 0.534	0.001	77.9	46.6, 90.8
	43+ days ago	—	—	—	—	—	—	—

Results are adjusted for age, sex, use of a long-lasting insecticidal net, socioeconomic status, and caregiver’s education, apart from Mali, 2015, where sex was not collected, and The Gambia, 2015, which is not adjusted for socioeconomic status, as this was missing for 162 records but was not an important confounder. Data on recent (non-SMC) antimalarial treatment was collected in 2016. In 3 countries recent antimalarial treatment was relatively rarely reported (6 times in The Gambia, 2016, and 34 times each in Burkina Faso, 2016, and Mali, 2016), and adjusting for recent treatment made very little difference to model estimates. In Chad, 2016, recent treatment with an antimalarial was commonly reported (17.1% of controls and 29.6% of cases). Adjustment for this in addition to the other covariates resulted in an estimate of PE of 73.4% (95% CI: 40.4%, 88.2%) in the first 28 days and 49.5% (95% CI: −47.7%, +82.7%) for the period 29–42 days. In Nigeria, 2016, recent treatment was reported by 3.74% of controls and 15.2% of cases, but adjusting for recent treatment made very little change to the estimates of PE: 81.2% (95% CI: 64.8%, 90.0%) in the first 28 days and 66.9% (95% CI: 17.2%, 86.8%) for the period 29–42 days. OR, odds ratio; PE, protective efficacy; SMC, seasonal malaria chemoprevention.

In 2016, the following numbers of case–control sets were recruited: 459 in Burkina Faso, 199 in Chad, 341 in Mali, 460 in Nigeria, and 248 in The Gambia. In Nigeria, 59 controls had dates of SMC recorded that were logically impossible (later than the date of recruitment), suggesting either issues with recording on the SMC card itself or with transcription onto the paper form; the 59 affected sets were discarded, leaving 401 case–control sets in the dataset. The number of discordant sets in which exposure differed between the case and controls was 95, 63, 194, 117, and 103, respectively, in Burkina Faso, Chad, Mali, Nigeria, and The Gambia (Table E in S1 Tables). After adjustment for age, sex, LLIN use, SES, and education, ORs for SMC within the previous 28 days ranged from 0.07 (95% CI: 0.03, 0.17) in Burkina Faso to 0.27 (95% CI: 0.18, 0.42) in Mali, indicating a range of protective effectiveness estimates between 72.9% and 93.4%. The OR and PE for SMC within 28 days by country were as follows: Burkina Faso, OR 0.07 (95% CI: 0.03, 0.17), PE 93.4% (95% CI: 83.1%, 97.4%); Chad, OR 0.22 (95% CI: 0.11, 0.47), PE 77.8% (95% CI: 53.1%, 89.5%); Mali, OR 0.27 (95% CI: 0.18, 0.42), PE 72.9% (95% CI: 58.5%, 82.3%); Nigeria, OR 0.17 (95% CI: 0.09, 0.31), PE 83.1% (95% CI: 69.1%, 90.8%); and The Gambia, OR 0.08 (95% CI: 0.04, 0.17), PE 91.9% (95% CI: 83.0%, 96.1%).

Protective effectiveness in the period 29–42 days ranged from 46.3% (95% CI: 8.2%, 68.6%) in Mali, to 77.9% (95% CI: 46.6%, 90.8%) in the Gambia (Table 2). PE in each country was as follows: Burkina Faso, 57.2% (95% CI: −14.4%, 84.0%); Chad, 56.6% (95% CI: −25.6%, 85.0%); Mali, 46.3% (95% CI: 8.2%, 68.6%); Nigeria, 63.7% (95% CI: 12.7%, 84.9%); and The Gambia, 77.9% (95% CI: 46.6%, 90.8%). As in 2015, in Mali there was no evidence of heterogeneity between Diema and Sanso districts in the association between malaria and SMC (LRT *p =* 0.81).

### Overall protective effectiveness of SMC against clinical malaria

**Fig 3:**
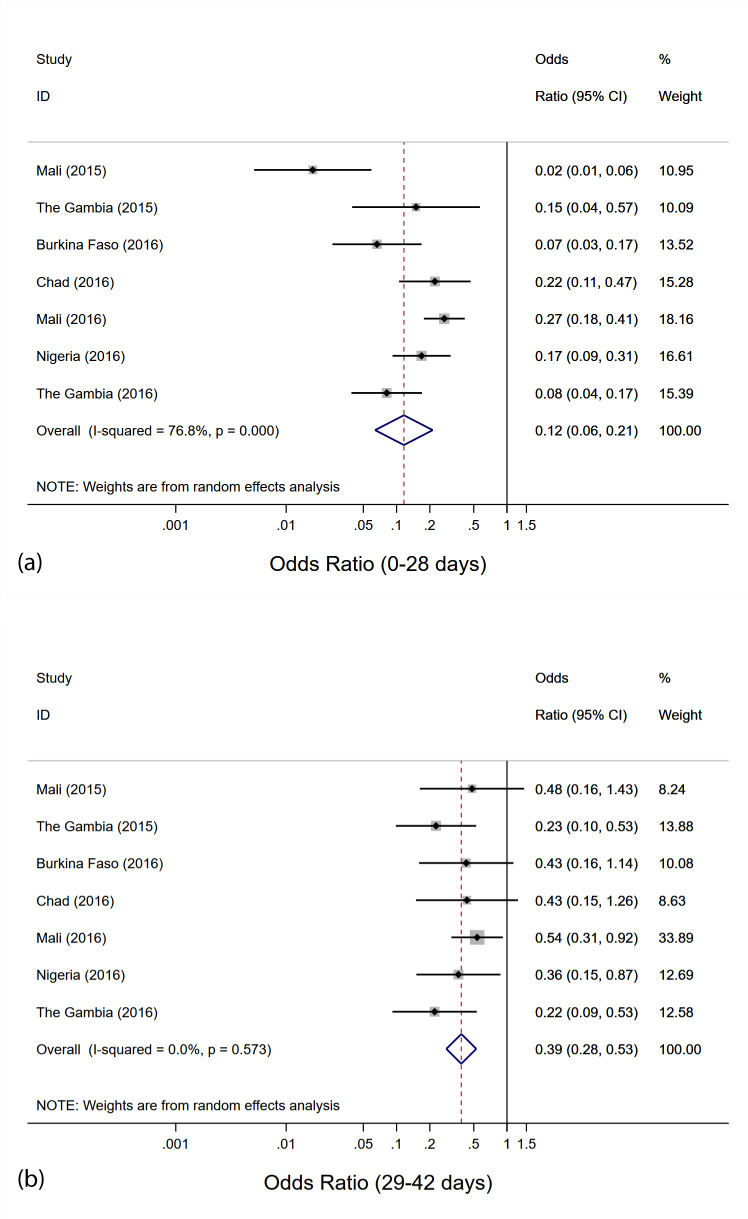
Meta-analysis of the odds ratio for seasonal malaria chemoprevention (SMC) within the previous 28 days and 29–42 days ago. (a) SMC within the previous 28 days; (b) SMC 29–42 days ago. Results from random-effects meta-analysis.

Overall, a random-effects meta-analysis including all 7 studies gave an estimated pooled OR of 0.12 (95% CI: 0.06, 0.21) for SMC in the previous 28 days, indicating a protective effectiveness of SMC against clinical malaria of 88% (95% CI: 79%, 94%) (Fig 3). This analysis indicated evidence of heterogeneity (LRT 25.9, 6 d.f., *I*^2^ 76.8% [95% CI: 51.5%, 88.9%]; *p <* 0.001) between studies. When the study from Mali, 2015, which had the highest estimate of protective effectiveness, was removed, there was then weaker evidence of heterogeneity (LRT 12.8, 5 d.f., *I*^2^ 60.9% [95% CI: 4.4%, 84.0%]; *p =* 0.025), and a pooled estimate of PE of 85% (95% CI: 76%, 95%). Effectiveness against clinical malaria in the period 29–42 days was 61% (95% CI: 47%, 72%), with no evidence for heterogeneity between studies (LRT 4.77, 6 d.f., *I*^2^ 0.0% [95% CI: 0.0%, 70.8%]; *p =* 0.57). The 90% prediction interval (which accounts for between-study variability) for the OR for SMC within the previous 28 days (i.e., where the effect size might be expected to lie in a future study) was 0.022 to 0.597, corresponding to a PE of 40.3% to 97.8%.

Fixed-effects meta-analysis gave a similar but slightly lower overall estimate of PE in the period 0–28 days: 84% (95% CI: 79%, 88%) (Fig B in S1 Fig). Use of fixed-effects meta-analysis did not change the estimated PE in the period 29–42 days: 61% (95% CI: 47%, 72%).

When the analysis was repeated restricted to matched case–control sets in which the case had parasite density above 5,000 per microlitre (and excluding Nigeria, 2016, where density was not estimated), the pooled OR was 0.10 (95% CI: 0.04, 0.21) for SMC in the previous 28 days, indicating a protective effectiveness of SMC against clinical malaria with high parasite density of 90% (95% CI: 79%, 96%), and 0.41 (95% CI: 0.26, 0.66) for SMC 29–42 days ago, indicating a protective effectiveness of 59% (95% CI: 34%, 74%) (Fig C in S1 Fig).

### Protective effectiveness of LLIN use against clinical malaria

Compared to SMC, there were fewer case–control pairs that were discordant for LLIN use, and consequently wide uncertainty in LLIN protective effectiveness in most studies (Tables F to L in S1 Tables for individual countries, summarised in Table M in S1 Tables). Protective effectiveness of LLIN use was demonstrated in The Gambia, 2015 (49.9% [95% CI: 19.1%, 69.0%]), and Nigeria, 2016 (75.1% [95% CI: 62.2%, 83.6%]).

## Discussion

Our results suggest that SMC provided a very high degree of personal protection against clinical malaria for the first 28 days after each treatment. Protection was then lower in the period 29–42 days after treatment, emphasising the importance of repeating treatments at monthly intervals. This finding was consistent across the different studies, which took place in locations representing a range of transmission intensity and seasonality patterns within the wider SMC area.

These results showing high protective effectiveness against clinical malaria are consistent with the results of previous clinical trials showing high protection from SMC [1], with reported good adherence to the 3-day SMC regimen (in this study and in the SMC coverage surveys conducted during ACCESS-SMC), and with the low frequency of molecular markers of resistance to SMC drugs observed in the implementing countries in West Africa [4]. It is reassuring that the profile of protection over time agrees well with that estimated from previous studies [17], and that consistent results were obtained with the more specific case definition of *P*. *falciparum* density of more than 5,000 per microlitre, for which malaria is more likely to be the true cause of fever. In large clinical trials of SP plus AQ for SMC [18,19], protective effectiveness against clinical malaria over the transmission season was 70% in Burkina Faso and 82% in Mali. These estimates are not directly comparable with estimates from the current case–control study as they included person-time at risk up to 6 weeks after the final SMC treatment, and included children who either missed SMC courses or had malaria at the time of SMC and received artemether–lumefantrine instead of SP plus AQ. The efficacy over 28 days in these trials was 83% [20], comparable to the effectiveness estimate in this study.

In this study, a child was considered to have received SMC if the first dose was administered by a community health worker, regardless of adherence to subsequent doses or vomiting of the medication. The high estimated effectiveness of SMC suggests that any failure to complete the 3-day regimen that may have occurred did not markedly reduce effectiveness.

It is challenging to measure adherence to the 3-day SMC regimen under routine conditions. Most caregivers reported that they completed the 3-day treatment course in this study, as was reported during coverage surveys during ACCESS-SMC [4]. Drug distributors are trained to repeat the first dose if the child vomits; administrative records suggest this occurs infrequently (e.g., K. M. Loua and P. Milligan, personal communication). However, it is common for young children to spit out some of the medication, and it is difficult to determine how much of the medication was ingested.

Large-scale molecular marker surveys were carried out in nearby areas of each country during the ACCESS-SMC project. Although the *dhfr*-triple mutations and the *dhps*-437 mutation (conferring resistance to pyrimethamine and sulphadoxine, respectively) are present in West Africa, the *dhps*-540 and *dhps*-581 mutations (which confer a higher grade resistance to SP) are very rare, and there is a low prevalence of the *mdr* and *pfcrt* mutations thought to confirm high-grade resistance to AQ [4]. Ongoing monitoring of these molecular markers will be needed to detect any increase in the prevalence and severity of SP resistance, but changes in the prevalence of markers alone cannot indicate in vivo efficacy of chemoprevention [5,6]. Case–control studies offer a reasonably quick and inexpensive approach to estimate protective effectiveness to complement this approach, and can be used to confirm effectiveness even in situations where impact is difficult to measure or interpret due to weak surveillance or changes in malaria detection rates or access to treatment.

There are a number of limitations to this study. There could be a potential bias towards higher estimated effectiveness of SMC against clinical malaria if malaria exposure is greater in areas with poorer access to SMC; selecting controls from the same neighbourhood attempts to at least partially control for this. Residual confounding may remain, but the sharp decrease in effectiveness with time since dose argues against a substantial bias. A potential bias towards lower estimated effectiveness of SMC is that cases might have better access to care than controls. If the factors that result in better access to care also result in better access to SMC, this might be associated with higher coverage of SMC among cases, biasing effectiveness downwards. Neighbourhood controls attempt to control for key factors affecting access, such as distance to health centre, but not all factors (e.g., caregivers with more or less time or resources to take children for treatment when unwell). Test-negative designs, which use patients who test negative for malaria as controls, would more fully control for access to care, but, in the Sahel context, where transmission remains high, selecting children without malaria parasitaemia (or non-febrile children with asymptomatic parasitaemia) would be likely to selectively include SMC recipients as controls. This would bias estimated effectiveness upwards, and thus neighbourhood controls were preferred.

Controls were more likely to be female than cases, and slightly older on average. However, adjustment for these factors suggests that this did not introduce important confounding. Children from households with a large number of other children may be under-represented in the controls, because control households were chosen first, then a single child within the household selected [21]. This could create an artefactual association between household size and malaria. However, household size did not appear to be associated with receipt of SMC, and controlling for household size did not change estimates of the association between malaria and SMC in any of the studies.

Previous exposure to SMC was documented based on the SMC card, where this was available, and otherwise according to caregiver recall, checked carefully against distribution dates for the community. Although there is the potential for this to introduce recall bias, provided controls are recruited at or soon after the time that the case is recruited, the length of the recall period is relatively short. Detailed coverage surveys carried out in other areas implementing SMC found reasonably high retention of the SMC distribution card, and good agreement between SMC card and recall within the same malaria transmission season [4].

Estimates of protection against clinical malaria within the first 28 days after SMC are likely to be sensitive to the exact timing of recruitment with respect to the previous SMC cycle. For example, the exposure status of cases could differ between the first week following SMC (when one would expect very few cases to have previously received SMC) and the final week of each monthly cycle (when more cases may have received SMC at the time of the last cycle, but still become unwell in spite of this). In 2016, we attempted to standardise this by recruiting a constant number of cases in each week of the study, over the whole season. In 2015, recruitment began towards the end of the malaria transmission season, and limiting the number of cases recruited in this way was not possible due to the declining malaria burden. This may explain the high efficacy estimate in Mali, in 2015, as many of the discordant case–control sets were recruited very soon after SMC (Fig A in S1 Fig), when the SP and AQ were likely to have very high efficacy.

Despite these limitations, the close agreement between the effectiveness estimates obtained in different locations within the SMC area, and in countries where different levels of SMC coverage were achieved (very high in Burkina Faso, and much lower in Nigeria and Chad [4]), is reassuring. Case–control studies have been widely used to evaluate preventive interventions such as vaccines [10], and other preventive interventions for malaria [12]. Use of case–control studies for the evaluation of SMC is more complicated than for interventions such as vaccines because (1) the expected duration of protection is short, even if SMC is working well, and (2) there are multiple SMC cycles within a short period of time. Both these factors mean that accurate documentation of the date of receipt of SMC is critical in a way that would not be the case for a vaccine providing lifelong immunity (for which prior receipt of the vaccine or not, as a binary outcome, may be sufficient, without reference to specific dates). Despite these challenges, our results suggest that this approach is a valid method to monitor the effectiveness of SMC in vivo, complementing other approaches, and potentially providing an approach to monitor the effect of SMC on severe malaria, which is difficult to do using prospective approaches. This approach also allows the effect of other risk factors to be estimated, including the protection from other interventions. We attempted to do this for LLINs in this study, but the high coverage of LLINs affected precision in some study centres.

Case–control studies require close attention to methodology, including quality-controlled microscopy at the point of recruitment and careful design, supervision, and analysis, and should to be undertaken by suitably trained teams. It is important to collect a full SMC history from all cases and controls, including SMC in previous months, and to measure potential confounders carefully. However, if these challenges can be met, the design can give meaningful results even when implemented in only a small number of health facilities, and the costs are relatively low, even with the need to travel to conduct home visits.

In areas where case–control studies are planned, there should be a particular effort to document SMC dates carefully, both on the SMC card and in SMC registers, and to record accurately the dates of SMC campaigns in the study area and the dates of any call back visits made by the delivery teams after the main distribution dates. In some countries implementing SMC (e.g., The Gambia), children have an ID card with a quick response (QR) code, permitting electronic data capture of SMC delivery, permitting more accurate ascertainment of treatment dates.

If carried out properly, case–control studies can be a powerful tool to monitor SMC effectiveness, and these studies should be repeated at regular intervals and in a range of locations. These studies could also be done reactively in response to concerns about low effectiveness (e.g., many children reporting malaria despite receipt of SMC) or apparent low impact (e.g., a particularly high burden in routine health system data). This is important because alarm about loss of effectiveness may arise from fluctuations in malaria incidence over time (leading to peaks in malaria cases that suggest loss of protection) and/or from the observation that a high percentage of malaria cases have recently received SMC (which would be expected if coverage is high) [22].

These results from 5 countries confirm that SMC as used in routine programmes provides a high degree of personal protection against clinical malaria, consistent with reported adherence and low frequencies of molecular markers of resistance, and support the continued deployment of SMC at scale to protect children in the Sahel and sub-Sahel regions of Africa.

## Supporting information

S1 STROBE Checklist (DOC)

S1 Fig Supplementary results Figs A to C, as referred to in the text. (DOCX)

S1 Methods Sample size for the case–control studies. (DOCX)

S1 Tables Supplementary results Tables A to M, as referred to in the text. (DOCX)

## Abbreviations

AQ: amodiaquine
LLIN: long-lasting insecticidal net
LRT: likelihood ratio test
OR: odds ratio
PC: personal computer
PE: protective efficacy
SMC: seasonal malaria chemoprevention
SES: socioeconomic status
SP: sulphadoxine–pyrimethamine
