# Supplementary material for: Effectiveness of seasonal malaria chemoprevention (SMC) treatments when SMC is implemented at scale: Case–control studies in 5 countries
Source: PLoS Med. 2021 Sep 8;18(9):e1003727. doi: 10.1371/journal.pmed.1003727 (PMC8457484; doi:10.1371/journal.pmed.1003727)
Supplement: S1 Fig — (DOCX) [file pmed.1003727.s002.docx]

**Figure A. Days since most recent SMC cycle for cases from discordant case-control sets, by study**

Days since most recent SMC course for cases from discordant case-control sets (i.e. where the time since the most recent SMC course was different for the case and at least one control). The Y-axis indicates the cumulative number of discordant pairs (for visualisation of the distribution only; exact numbers are given in Supplementary Table S5). Vertical dashed lines show the 28 day and 42 day categories used in the regression models. Among the discrepant case-control sets, it was generally rare for the case to have received SMC recently (shown by the small number of data points before 28 days), and more common for cases to have received SMC a long time ago (shown by the larger number of data points beyond 28 days, beyond 42 days, and particularly ‘no SMC’ – indicating no SMC yet in the current implementation year.

**Figure B. Fixed Effects meta-analysis of the odds ratio for SMC within the previous 28 days and between 29-42 days ago.**

**
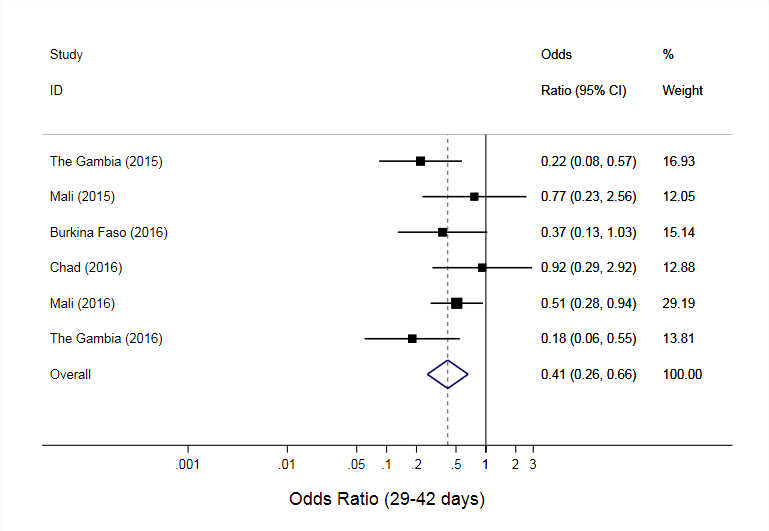
Figure C. Random-effects meta-analysis plots for protection from SMC between 0-28 days and 29-42 days, restricted to case-control sets where the case had parasite density > 5000 per ul.**


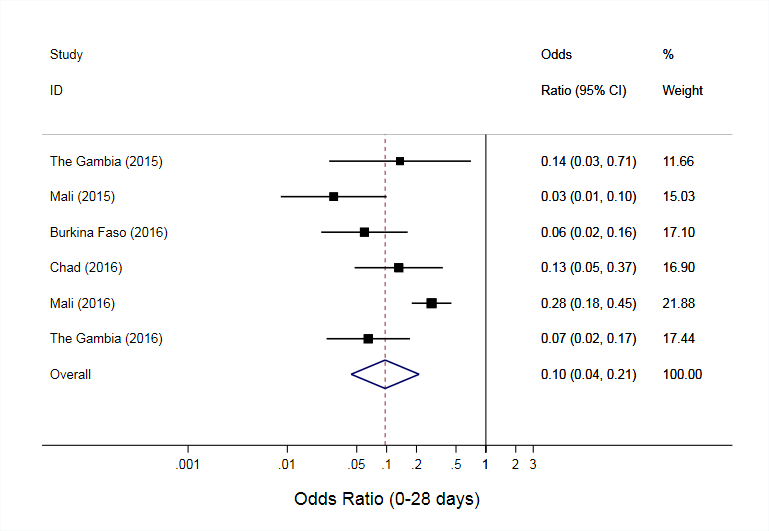


$ In Nigeria, 2016, a coding system was used for parasite density, and exact density cannot be estimated.
