# Supplementary material for: Effectiveness of seasonal malaria chemoprevention (SMC) treatments when SMC is implemented at scale: Case–control studies in 5 countries
Source: PLoS Med. 2021 Sep 8;18(9):e1003727. doi: 10.1371/journal.pmed.1003727 (PMC8457484; doi:10.1371/journal.pmed.1003727)
Supplement: S1 Methods — (DOCX) [file pmed.1003727.s003.docx]

**S1 Methods. Sample size for the case-control studies**

In a matched case-control study with 2 controls per case, if SMC coverage is 90%, 134 cases are required for 80% power to detect an efficacy of 70%, and 202 cases for 95% power. In a previous case control study of SMC (Bojang et al., PLoS Medicine, 2011; doi:10.1371/journal.pmed.1000409), the monthly coverage of SMC ranged from 62% to 92%, and there were 81 cases and 89 controls. This yielded an estimate of efficacy of 80%, with a 95% confidence interval 42% to 93%. We wished to estimate efficacy of SMC during 28 days post-treatment and efficacy between 29 and 42 days post treatment. Case-control pairs were therefore to be enrolled each week during the 4 months of the SMC period and during the 4 subsequent weeks to ensure there were matched sets in different periods post treatment. A total sample size of about 240 would be needed in each country to ensure that about 12 cases could be enrolled each week, this would give about 192 cases during the main 4-month intervention period, sufficient to estimate efficacy of SMC over 28 days allowing for losses due to missing information or failure to recruit both controls and adjustment for confounders.
