# Supplementary material for: Effectiveness of seasonal malaria chemoprevention (SMC) treatments when SMC is implemented at scale: Case–control studies in 5 countries
Source: PLoS Med. 2021 Sep 8;18(9):e1003727. doi: 10.1371/journal.pmed.1003727 (PMC8457484; doi:10.1371/journal.pmed.1003727)
Supplement: S1 Tables — (DOCX) [file pmed.1003727.s004.docx]

**Table A.** Time interval between recruitment of case and recruitment of controls, by study

|  | **Mali 2015** | | **Gambia 2015** | | **Burkina Faso 2016** | | **Chad 2016** | | **Mali 2016** | | **Nigeria 2016** | | **Gambia 2016** | |
| --- | --- | --- | --- | --- | --- | --- | --- | --- | --- | --- | --- | --- | --- | --- |
| Days since case | n | % | n | % | n | % | n | % | n | % | n | % | n | % |
| 0 | 338 | 67.1 | 434 | 96.2 | 174 | 19.0 | 191 | 48.0 | 638 | 93.6 | 256 | 31.9 | 303 | 61.1 |
| 1 | 137 | 27.2 | 5 | 1.1 | 168 | 18.3 | 61 | 15.3 | 36 | 5.3 | 181 | 22.6 | 93 | 18.8 |
| 2 | 16 | 3.2 | 0 | 0.0 | 121 | 13.2 | 45 | 11.3 | 2 | 0.3 | 175 | 21.8 | 44 | 8.9 |
| 3 | 3 | 0.6 | 0 | 0.0 | 69 | 7.5 | 8 | 2.0 | 0 | 0.0 | 85 | 10.6 | 23 | 4.6 |
| 4 | 0 | 0.0 | 0 | 0.0 | 46 | 5.0 | 22 | 5.5 | 0 | 0.0 | 42 | 5.2 | 10 | 2.0 |
| 5 | 0 | 0.0 | 0 | 0.0 | 43 | 4.7 | 14 | 3.5 | 0 | 0.0 | 25 | 3.1 | 11 | 2.2 |
| 6 | 0 | 0.0 | 0 | 0.0 | 15 | 1.6 | 8 | 2.0 | 0 | 0.0 | 17 | 2.1 | 10 | 2.0 |
| 7 | 0 | 0.0 | 0 | 0.0 | 8 | 0.9 | 6 | 1.5 | 0 | 0.0 | 7 | 0.9 | 2 | 0.4 |
| >7 & <=30 days | 0 | 0.0 | 12 | 2.7 | 209 | 22.8 | 42 | 10.6 | 0 | 0.0 | 0 | 0.0 | 0 | 0.0 |
| missing* | 10 | 2.0 | 0 | 0.0 | 65 | 7.1 | 0 | 0.0 | 6 | 0.9 | 14 | 1.8 | 0 | 0.0 |

**Table B**. Percentage of children where the SMC card was seen at the home visit.

|  |  | Mali, 2015 | | The Gambia, 2015 | | Burkina Faso, 2016 | | Chad, 2016 | | Mali, 2016 | | Nigeria, 2016 | | The Gambia, 2016 | |
| --- | --- | --- | --- | --- | --- | --- | --- | --- | --- | --- | --- | --- | --- | --- | --- |
|  |  | Controls | Cases | Controls | Cases | Controls | Cases | Controls | Cases | Controls | Cases | Controls | Cases | Controls | Cases |
|  |  | N=504, % | N=252, % | N= 452, % | N = 226, % | N=918, % | N=459, % | N=398, % | N=199, % | N=682, % | N=341, % | N=802, % | N=401, % | N=496, % | N=248, % |
|  |  |  |  |  |  |  |  |  |  |  |  |  |  |  |  |
| SMC card | No | 46.1 | 70.2 | 8.1 | 31.5 | 0.7 | 0.2 | 33.2 | 56.8 | 65.8 | 75.7 | 26.1 | 38.9 | 29.8 | 46.0 |
| seen | Yes | 53.9 | 29.8 | 91.9 | 68.5 | 99.4 | 99.8 | 66.8 | 43.2 | 34.0 | 24.3 | 73.9 | 61.1 | 70.2 | 54.0 |
|  | n missing | 1 | 0 | 8 | 4 | - | - | - | - | 1 | - | - | - | - | - |

**Table C. Parasite density for malaria cases in each study**

| Country, Year | No. cases | No. cases (%) with parasitaemia >5000/u l | Geometric mean parasite density (95% CI) |
| --- | --- | --- | --- |
| Mali, 2015 | 252 | 196 (77.8) | 20,526.9 (16,017.3, 26,306.2) |
| The Gambia, 2015 | 226 | 153 (67.7) | 10,322.6 (7,887.4, 13,509.5 |
|  |  |  |  |
| Burkina Faso, 2016 | 459 | 372 (81.1) | 19,635.7 (16,151.1, 23,872.0) |
| Chad, 2016 | 199 | 131 (65.8) | 31,596.4 (22,699.7, 43,980.1) |
| Mali, 2016 | 341 | 284 (83.3) | 24,191.4 (19,398.8, 30,168.1) |
| Nigeria, 2016^$^ |  | NA | NA |
| The Gambia, 2016 | 248 | 173 (69.8) | 9,475.7 (7,372.4, 12,179.1) |

^$^ In Nigeria, 2016, a coding system was used for parasite density, and exact density cannot be estimated.

**Table D. Caregiver-reported adherence to SMC daily dosing among cases and controls on the most recent occasion that SMC was administered to the child**

|  |  | **Controls** |  | **Cases** |  | **Total** |
| --- | --- | --- | --- | --- | --- | --- |
| **All 3 doses administered** | n | % | n | % | n | % |
| Mali-2015 | 357 | 99.2 | 113 | 95.0 | 470 | 98.1 |
| The Gambia-2015 | 420 | 96.1 | 175 | 89.7 | 595 | 94.1 |
| Burkina-2016 | 909 | 99.7 | 453 | 99.8 | 1362 | 99.7 |
| Chad-2016 | 263 | 98.5 | 89 | 97.8 | 352 | 98.3 |
| Mali-2016 | 456 | 94.4 | 187 | 96.9 | 643 | 95.1 |
| Nigeria-2016 | 670 | 99.7 | 267 | 97.8 | 937 | 99.2 |
| The Gambia-2016 | 446 | 94.9 | 205 | 96.2 | 651 | 95.3 |
|  |  |  |  |  |  |  |
| **Swallowed all 3 daily doses with no issues** | n | % | n | % | n | % |
| Mali-2015 | 299 | 83.1 | 89 | 74.8 | 388 | 81.0 |
| The Gambia-2015 | 270 | 61.8 | 65 | 33.3 | 335 | 53.0 |
| Burkina-2016 | 886 | 97.1 | 432 | 95.2 | 1318 | 96.5 |
| Chad-2016 | 216 | 80.9 | 72 | 79.1 | 288 | 80.4 |
| Mali-2016 | 369 | 76.4 | 116 | 60.1 | 485 | 71.7 |
| Nigeria-2016 | 583 | 86.8 | 237 | 86.8 | 820 | 86.8 |
| The Gambia-2016 | 342 | 72.8 | 54 | 25.4 | 396 | 58.0 |
|  |  |  |  |  |  |  |
| **Vomited or spat one or more doses** | n | % | n | % | n | % |
| Mali-2015 | 57 | 15.8 | 25 | 21.0 | 82 | 17.1 |
| The Gambia-2015 | 151 | 34.6 | 124 | 63.6 | 275 | 43.5 |
| Burkina-2016 | 22 | 2.4 | 19 | 4.2 | 41 | 3.0 |
| Chad-2016 | 48 | 18.0 | 17 | 18.7 | 65 | 18.2 |
| Mali-2016 | 95 | 19.7 | 74 | 38.3 | 169 | 25.0 |
| Nigeria-2016 | 49 | 7.3 | 19 | 7.0 | 68 | 7.2 |
| The Gambia-2016 | 112 | 23.8 | 153 | 71.8 | 265 | 38.8 |
|  |  |  |  |  |  |  |
| **Child refused one or more dose of SMC** | n | % | n | % | n | % |
| Mali-2015 | 1 | 0.3 | 0 | 0.0 | 1 | 0.2 |
| The Gambia-2015 | 4 | 0.9 | 1 | 0.5 | 5 | 0.8 |
| Burkina-2016 | 1 | 0.1 | 0 | 0.0 | 1 | 0.1 |
| Chad-2016 | 8 | 3.0 | 4 | 4.4 | 12 | 3.4 |
| Mali-2016 | 3 | 0.6 | 2 | 1.0 | 5 | 0.7 |
| Nigeria-2016 | 39 | 5.8 | 14 | 5.1 | 53 | 5.6 |
| The Gambia-2016 | 2 | 0.4 | 1 | 0.5 | 3 | 0.4 |

The first section of the table shows, on the most recent occasion SMC was administered to the child, whether all three daily doses of SMC (SP+AQ on day 1, and AQ alone on days 2 and 3) were administered. The second section shows whether all 3 daily doses were swallowed without any problems. The third section shows whether vomiting or spitting* of medication was reported on any of the three days. The fourth section shows whether any of the daily doses were refused. The denominator for all these sections is children who had previously received SMC, irrespective of the time frame, since those without previous SMC could not answer these questions. *Vomiting or spitting of medication may be hard to distinguish in practice: systemic vomiting after administration of AQ does occur, but is rare. It is much more common for children to spit out some of the medication in response to the bitter taste. Determining the difference between these possibilities is challenging, particularly in retrospect.

**Table E. Discrepancy in exposure to recent SMC among the case-control sets**

|  |  | **No. of controls with discrepant exposure within set** | | | | | |
| --- | --- | --- | --- | --- | --- | --- | --- |
|  |  | **2** | **1** | **0** | **1 concordant,**  **1 missing exposure** | **Both controls missing exposure** | **Total** |
| **Study** | **Case's exposure to SMC** |  |  |  |  |  |  |
|  |  |  |  |  |  |  |  |
| **Mali** | SMC within 28 days | 0 | 3 | 10 | 2 | 0 | 15 |
| **(2015)** | SMC 29-42 days | 9 | 2 | 8 | 1 | 0 | 20 |
|  | SMC 43+ days ago | 63 | 34 | 52 | 15 | 21 | 185 |
|  | missing |  |  |  |  |  | 32 |
|  | Total | 72 | 39 | 70 | 18 | 21 | 252 |
|  |  |  |  |  |  |  |  |
| **The** | SMC within 28 days | 0 | 3 | 13 | 2 | 0 | 18 |
| **Gambia** | SMC 29-42 days | 1 | 7 | 12 | 0 | 0 | 20 |
| (**2015)** | SMC 43+ days ago | 23 | 17 | 138 | 4 | 1 | 183 |
|  | missing |  |  |  |  |  | 5 |
|  | Total | 24 | 27 | 163 | 6 | 1 | 226 |
|  |  |  |  |  |  |  |  |
| **Burkina** | SMC within 28 days | 5 | 7 | 261 | 0 | 0 | 273 |
| **Faso** | SMC 29-42 days | 34 | 4 | 77 | 0 | 0 | 115 |
| **(2016)** | SMC 43+ days ago | 44 | 1 | 26 | 0 | 0 | 71 |
|  | Total | 83 | 12 | 364 | 0 | 0 | 459 |
|  |  |  |  |  |  |  |  |
| **Chad** | SMC within 28 days | 7 | 6 | 11 | 0 | 0 | 24 |
| **(2016)** | SMC 29-42 days | 5 | 2 | 3 | 0 | 0 | 10 |
|  | SMC 43+ days ago | 24 | 19 | 122 | 0 | 0 | 165 |
|  | Total | 36 | 27 | 136 | 0 | 0 | 199 |
|  |  |  |  |  |  |  |  |
| **Mali** | SMC within 28 days | 13 | 29 | 26 | 0 | 0 | 68 |
| **(2016)** | SMC 29-42 days | 21 | 11 | 4 | 1 | 0 | 37 |
|  | SMC 43+ days ago | 65 | 55 | 113 | 1 | 0 | 234 |
|  | missing |  |  |  |  |  | 2 |
|  | Total | 99 | 95 | 143 | 2 | 0 | 341 |
|  |  |  |  |  |  |  |  |
| **Nigeria** | SMC within 28 days | 13 | 11 | 110 | 7 | 6 | 147 |
| **(2016)** | SMC 29-42 days | 10 | 6 | 33 | 1 | 0 | 50 |
|  | SMC 43+ days ago | 48 | 29 | 123 | 0 | 4 | 204 |
|  | Total | 71 | 46 | 266 | 8 | 10 | 401 |
|  |  |  |  |  |  |  |  |
| **The** | SMC within past 28 days | 2 | 14 | 126 | 0 | 0 | 142 |
| **Gambia** | SMC 29-42 days ago | 16 | 5 | 14 | 0 | 0 | 35 |
| **(2016)** | SMC 43+ days ago | 51 | 15 | 5 | 0 | 0 | 71 |
|  | Total | 69 | 34 | 145 | 0 | 0 | 248 |

The table shows the exposure status of the cases (how long ago SMC was received), and the number of controls with a *different* exposure status. If there was no missing data on exposure, then the possible values are 2, 1 or 0 controls with the same exposure as the case. The left-hand most columns headed ‘2’ or ‘1’ indicate that either 2 controls or 1 control, respectively, had a *different* exposure status to the case, and these case-control sets are therefore informative as to the effect of SMC. For example, in Mali (2015), there are 72 + 39 = 111 discrepant case-control sets that contribute to the estimate of SMC efficacy, from 252 case-control sets recruited. The column headed ‘0’ indicates that the case and both controls had the same exposure status: there are no discrepancies, and therefore these case-control sets are not informative. The columns ‘1 concordant, 1 missing exposure’ and ‘both controls missing exposure’ indicate the situations where one or both controls were missing their exposure status.

**Table F. Mali 2015 - Results of crude and multivariable conditional logistic regression**

|  | **Crude OR** | **95% CI** | **Adjusted OR** | **95% CI** | **P-value** | **PE %** | **95% CI** |
| --- | --- | --- | --- | --- | --- | --- | --- |
| **SMC** |  |  |  |  |  |  |  |
| within past 28 days | 0.022 | (0.007, 0.070) | 0.017 | (0.005, 0.059) | <0.001 | 98.3 | (94.1, 99.5) |
| 29-42 days ago | 0.47 | (0.17, 1.31) | 0.48 | (0.16, 1.43) | 0.189 | 51.8 | (-43.2, 83.8) |
| 43+ days ago | - | - | - | - | - | - | - |
|  |  |  |  |  |  |  |  |
| **Age** |  |  |  |  | **<0.001*** |  |  |
| under 1 year | 0.14 | (0.07, 0.31) | 0.07 | (0.02, 0.26) | <0.001 | 92.9 | (74.1, 98.1) |
| 1 year | 0.46 | (0.27, 0.78) | 0.39 | (0.18, 0.83) | 0.015 | 61.2 | (17.0, 81.9) |
| 2 years | 0.57 | (0.36, 0.92) | 0.59 | (0.27, 1.25) | 0.167 | 41.5 | (-25.2, 72.7) |
| 3 years | 0.69 | (0.46, 1.04) | 0.69 | (0.39, 1.21) | 0.195 | 31.1 | (-21.1, 60.8) |
| 4 years | - | - | - | - | - | - | - |
|  |  |  |  |  |  |  |  |
| **LLIN** |  |  |  |  |  |  |  |
| yes | 1.00 | (0.33, 3.04) | 0.32 | (0.03, 3.27) | 0.335 | 68.2 | (-226.6, 96.9) |
| no | - | - | - | - | - | - | - |
|  |  |  |  |  |  |  |  |
| **SES** |  |  |  |  | **0.787*** |  |  |
| Highest | - | - | - | - | - | - | - |
| High | 0.74 | (0.44, 1.22) | 0.66 | (0.32, 1.36) | 0.256 | 34.5 | (-36.0, 68.5) |
| Medium | 0.64 | (0.37, 1.11) | 0.69 | (0.32, 1.46) | 0.330 | 31.4 | (-46.4, 67.9) |
| Low | 0.69 | (0.40, 1.20) | 0.74 | (0.35, 1.55) | 0.423 | 26.3 | (-55.4, 65.0) |
| Lowest | 0.54 | (0.30, 0.97) | 0.66 | (0.29, 1.46) | 0.302 | 34.5 | (-46.3, 70.7) |
|  |  |  |  |  |  |  |  |
| **Education** |  |  |  |  | **0.097*** |  |  |
| None | - | - | - | - | - | - | - |
| Koranic | 0.83 | (0.44, 1.54) | 1.17 | (0.49, 2.80) | 0.729 | -16.8 | (-180.3, 51.4) |
| Primary | 1.34 | (0.84, 2.14) | 1.00 | (0.45, 2.21) | 0.994 | 0.3 | (-121.1, 55.0) |
| Secondary / College | 0.51 | (0.27, 0.97) | 0.32 | (0.12, 0.85) | 0.022 | 67.7 | (15.0, 87.7) |

The reference category for each variable is indicated with a dash (-). For the categorical variables Age, Socio-economic Status (SES) and Education, the Wald Test P-values are provided in the rows for the specific categories, and the P-value from the global Likelihood Ratio Test for the overall association of the variable is also provided, marked with an asterisk (*), in the heading row.

**Table G. The Gambia 2015 - Results of crude and multivariable conditional logistic regression**

|  | **Crude OR** | **95% CI** | **Adjusted OR** | **95% CI** | **P-value** | **PE %** | **95% CI** |
| --- | --- | --- | --- | --- | --- | --- | --- |
| **SMC** |  |  |  |  |  |  |  |
| within past 28 days | 0.131 | (0.035, 0.487) | 0.150 | (0.040, 0.569) | 0.005 | 85.0 | (43.1, 96.0) |
| 29-42 days ago | 0.21 | (0.09, 0.47) | 0.23 | (0.10, 0.53) | 0.001 | 77.2 | (47.3, 90.2) |
| 43+ days ago | - | - | - | - | - | - | - |
|  |  |  |  |  |  |  |  |
| **Sex** |  |  |  |  |  |  |  |
| Female | - | - | - | - | - | - | - |
| Male | 0.85 | (0.62, 1.17) | 0.86 | (0.61, 1.23) | 0.409 | 13.7 | (-22.5, 39.2) |
|  |  |  |  |  |  |  |  |
| **Age** |  |  |  |  | **0.004*** |  |  |
| under 1 year | 0.27 | (0.10, 0.70) | 0.24 | (0.09, 0.67) | 0.006 | 75.6 | (32.6, 91.2) |
| 1 year | 0.43 | (0.25, 0.73) | 0.44 | (0.25, 0.78) | 0.005 | 56.1 | (21.8, 75.3) |
| 2 years | 0.76 | (0.47, 1.24) | 0.77 | (0.45, 1.30) | 0.321 | 23.4 | (-29.7, 54.7) |
| 3 years | 0.96 | (0.60, 1.54) | 0.91 | (0.55, 1.53) | 0.727 | 8.8 | (-52.7, 45.5) |
| 4 years | - | - | - | - | - | - | - |
|  |  |  |  |  |  |  |  |
| **LLIN** |  |  |  |  |  |  |  |
| yes | 0.42 | (0.27, 0.66) | 0.50 | (0.31, 0.81) | 0.005 | 49.9 | (19.1, 69.0) |
| no | - | - | - | - | - | - | - |
|  |  |  |  |  |  |  |  |
| **SES** |  |  |  |  |  |  |  |
| Highest | - | - | - | - | - | - | - |
| High | 0.75 | (0.41, 1.39) |  |  |  |  |  |
| Medium | 0.73 | (0.39, 1.36) | [SES not included in multivariable model] | | | | |
| Low | 0.58 | (0.28, 1.18) |  |  |  |  |  |
| Lowest | 0.47 | (0.22, 1.00) |  |  |  |  |  |
|  |  |  |  |  |  |  |  |
| **Education** |  |  |  |  | **0.978*** |  |  |
| None | - | - | - | - | - | - | - |
| Koranic | 0.75 | (0.42, 1.34) | 0.93 | (0.48, 1.81) | 0.831 | 7.0 | (-80.6, 52.1) |
| Primary or higher | 0.70 | (0.35, 1.39) | 0.87 | (0.40, 1.90) | 0.727 | 13.0 | (-89.7, 60.1) |

The reference category for each variable is indicated with a dash (-). For the categorical variables Age and Education, the Wald Test P-values are provided in the rows for the specific categories, and the P-value from the global Likelihood Ratio Test for the overall association of the variable is also provided, marked with an asterisk (*), in the heading row. SES was not included in the multivariable model because this information was missing for 162 children and did not appear to be an important confounder.

**Table H. Burkina Faso 2016 - Results of crude and multivariable conditional logistic regression**

|  | **Crude OR** | **95% CI** | **Adjusted OR** | **95% CI** | **P-value** | **PE %** | **95% CI** |
| --- | --- | --- | --- | --- | --- | --- | --- |
| **SMC** |  |  |  |  |  |  |  |
| within past 28 days | 0.047 | (0.020, 0.113) | 0.066 | (0.026, 0.169) | <0.001 | 93.4 | (83.1, 97.4) |
| 29-42 days ago | 0.31 | (0.13, 0.74) | 0.43 | (0.16, 1.14) | 0.091 | 57.2 | (-14.4, 84.0) |
| 43+ days ago | - | - | - | - | - | - | - |
|  |  |  |  |  |  |  |  |
| **Sex** |  |  |  |  |  |  |  |
| Female | - | - | - | - | - | - | - |
| Male | 0.90 | (0.72, 1.13) | 0.97 | (0.75, 1.25) | 0.790 | 3.4 | (-24.8, 25.3) |
|  |  |  |  |  |  |  |  |
| **Age** |  |  |  |  | **0.527*** |  |  |
| under 1 year | 0.91 | (0.39, 2.09) | 1.12 | (0.40, 3.12) | 0.830 | -11.9 | (-212.4, 59.9) |
| 1 year | 0.68 | (0.37, 1.25) | 0.66 | (0.31, 1.37) | 0.263 | 34.4 | (-37.4, 68.7) |
| 2 years | 0.78 | (0.44, 1.37) | 0.76 | (0.39, 1.50) | 0.435 | 23.6 | (-50.1, 61.1) |
| 3 years | 0.82 | (0.48, 1.39) | 0.69 | (0.37, 1.30) | 0.254 | 30.8 | (-30.2, 63.2) |
| 4 years | - | - | - | - | - | - | - |
|  |  |  |  |  |  |  |  |
| **LLIN** |  |  |  |  |  |  |  |
| no | - | - | - | - | - | - | - |
| yes | 0.35 | (0.20, 0.63) | 0.53 | (0.27, 1.04) | 0.066 | 47.4 | (-4.4, 73.5) |
|  |  |  |  |  |  |  |  |
| **SES** |  |  |  |  | **<0.001*** |  |  |
| Highest | - | - | - | - | - | - | - |
| High | 4.05 | (2.41, 6.80) | 2.72 | (1.53, 4.83) | 0.001 | -171.6 | (-382.7, -52.8) |
| Medium | 12.98 | (7.09, 23.77) | 6.87 | (3.52, 13.4) | 0.000 | -586.8 | (-1241.3, -251.7) |
| Low | 10.14 | (5.30, 19.39) | 5.23 | (2.55, 10.70) | <0.001 | -422.6 | (-970.2, -155.2) |
| Lowest | 5.65 | (2.82, 11.31) | 3.25 | (1.53, 6.89) | 0.002 | -224.7 | (-588.7, -53.1) |
|  |  |  |  |  |  |  |  |
| **Education** |  |  |  |  | **0.009*** |  |  |
| None | - | - | - | - | - | - | - |
| Primary / Koranic | 0.63 | (0.43, 0.90) | 0.99 | (0.64, 1.54) | 0.968 | 0.9 | (-54.3, 36.4) |
| Secondary / College | 0.25 | (0.15, 0.43) | 0.40 | (0.21, 0.76) | 0.005 | 59.8 | (24.0, 78.7) |

The reference category for each variable is indicated with a dash (-). For the categorical variables Age, Socio-economic Status (SES) and Education, the Wald Test P-values are provided in the rows for the specific categories, and the P-value from the global Likelihood Ratio Test for the overall association of the variable is also provided, marked with an asterisk (*), in the heading row.

**Table I. Chad 2016 - Results of crude and multivariable conditional logistic regression**

|  | **Crude OR** | **95% CI** | **Adjusted OR** | **95% CI** | **P-value** | **PE %** | **95% CI** |
| --- | --- | --- | --- | --- | --- | --- | --- |
| **SMC** |  |  |  |  |  |  |  |
| within past 28 days | 0.317 | (0.163, 0.618) | 0.222 | (0.105, 0.469) | <0.001 | 77.8 | (53.1, 89.5) |
| 29-42 days ago | 0.44 | (0.17, 1.16) | 0.43 | (0.15, 1.26) | 0.124 | 56.6 | (-25.6, 85.0) |
| 43+ days ago | - | - | - | - | - | - | - |
|  |  |  |  |  |  |  |  |
| **Sex** |  |  |  |  |  |  |  |
| Female | - | - | - | - | - | - | - |
| Male | 0.96 | (0.67, 1.37) | 1.02 | (0.69, 1.52) | 0.907 | -2.4 | (-52.4, 31.2) |
|  |  |  |  |  |  |  |  |
| **Age** |  |  |  |  | **<0.001*** |  |  |
| under 1 year | 0.29 | (0.15, 0.56) | 0.25 | (0.12, 0.51) | <0.001 | 75.5 | (48.9, 88.2) |
| 1 year | 0.26 | (0.14, 0.49) | 0.23 | (0.12, 0.45) | <0.001 | 77.3 | (55.2, 88.5) |
| 2 years | 0.27 | (0.15, 0.48) | 0.27 | (0.14, 0.50) | <0.001 | 73.5 | (50.1, 85.9) |
| 3 years | 0.32 | (0.17, 0.63) | 0.33 | (0.16, 0.67) | 0.002 | 66.9 | (33.1, 83.6) |
| 4 years | - | - | - | - | - | - | - |
|  |  |  |  |  |  |  |  |
| **LLIN** |  |  |  |  |  |  |  |
| no | - | - | - | - | - | - | - |
| yes | 1.46 | (0.92, 2.32) | 1.33 | (0.80, 2.22) | 0.275 | -33.0 | (-122.1, 20.3) |
|  |  |  |  |  |  |  |  |
| **SES** |  |  |  |  | **<0.001*** |  |  |
| Highest | - | - | - | - | - |  |  |
| High | 0.98 | (0.49, 1.97) | 0.79 | (0.38, 1.65) | 0.529 | 21.0 | (-64.7, 62.1) |
| Medium | 2.33 | (1.13, 4.80) | 1.96 | (0.91, 4.23) | 0.086 | -96.1 | (-323.1, 9.6) |
| Low | 2.48 | (1.13, 5.46) | 2.72 | (1.19, 6.25) | 0.018 | -172.2 | (-525.0, -18.5) |
| Lowest | 3.02 | (1.43, 6.40) | 3.37 | (1.52, 7.47) | 0.003 | -237.1 | (-647.1, -52.1) |
|  |  |  |  |  |  |  |  |
| **Education** |  |  |  |  | **0.014*** |  |  |
| None | - | - | - | - | - | - | - |
| Koranic | 0.88 | (0.41, 1.89) | 1.01 | (0.43, 2.38) | 0.979 | -1.2 | (-138.0, 57.0) |
| Primary | 0.48 | (0.23, 0.98) | 0.46 | (0.21, 1.02) | 0.055 | 54.2 | (-1.5, 79.4) |
| Secondary / College | 0.60 | (0.29, 1.27) | 0.66 | (0.29, 1.52) | 0.333 | 33.7 | (-52.4, 71.2) |

The reference category for each variable is indicated with a dash (-). For the categorical variables Age, Socio-economic Status (SES) and Education, the Wald Test P-values are provided in the rows for the specific categories, and the P-value from the global Likelihood Ratio Test for the overall association of the variable is also provided, marked with an asterisk (*), in the heading row.

**Table J. Mali 2016 - Results of crude and multivariable conditional logistic regression**

|  | **Crude OR** | **95% CI** | **Adjusted OR** | **95% CI** | **P-value** | **PE %** | **95% CI** |
| --- | --- | --- | --- | --- | --- | --- | --- |
| **SMC** |  |  |  |  |  |  |  |
| within past 28 days | 0.294 | (0.201, 0.431) | 0.271 | (0.177, 0.415) | <0.001 | 72.9 | (58.5, 82.3) |
| 29-42 days ago | 0.64 | (0.39, 1.04) | 0.54 | (0.31, 0.92) | 0.023 | 46.3 | (8.2, 68.6) |
| 43+ days ago | - | - | - | - | - | - | - |
|  |  |  |  |  |  |  |  |
| **Sex** |  |  |  |  |  |  |  |
| Female | - | - | - | - | - | - | - |
| Male | 0.85 | (0.65, 1.11) | 0.81 | (0.60, 1.09) | 0.155 | 19.5 | (-8.6, 40.3) |
|  |  |  |  |  |  |  |  |
| **Age** |  |  |  |  | **<0.001*** |  |  |
| under 1 year | 0.16 | (0.08, 0.31) | 0.16 | (0.08, 0.32) | <0.001 | 84.0 | (68.1, 92.0) |
| 1 year | 0.36 | (0.23, 0.56) | 0.37 | (0.23, 0.61) | <0.001 | 62.8 | (39.1, 77.3) |
| 2 years | 0.95 | (0.66, 1.37) | 1.07 | (0.72, 1.61) | 0.737 | -7.2 | (-60.5, 28.4) |
| 3 years | 1.04 | (0.72, 1.49) | 1.20 | (0.81, 1.78) | 0.365 | -19.9 | (-77.6, 19.1) |
| 4 years | - | - | - | - | - | - | - |
|  |  |  |  |  |  |  |  |
| **LLIN** |  |  |  |  |  |  |  |
| no | - | - | - | - | - | - | - |
| yes | 1.19 | (0.52, 2.69) | 1.28 | (0.51, 3.20) | 0.605 | -27.6 | (-220.4, 49.2) |
|  |  |  |  |  |  |  |  |
| **SES** |  |  |  |  | **0.824*** |  |  |
| Highest | - | - | - | - | - | - | - |
| High | 0.94 | (0.60, 1.46) | 0.97 | (0.59, 1.59) | 0.891 | 3.4 | (-58.8, 41.3) |
| Medium | 0.94 | (0.59, 1.50) | 1.07 | (0.64, 1.80) | 0.795 | -7.1 | (-79.5, 36.1) |
| Low | 0.85 | (0.53, 1.35) | 0.90 | (0.54, 1.50) | 0.696 | 9.7 | (-50.4, 45.7) |
| Lowest | 0.73 | (0.43, 1.25) | 0.78 | (0.43, 1.42) | 0.407 | 22.4 | (-41.5, 57.5) |
|  |  |  |  |  |  |  |  |
| **Education** |  |  |  |  | **0.527*** |  |  |
| None | - | - | - | - | - | - | - |
| Koranic | 0.92 | (0.55, 1.54) | 0.74 | (0.42, 1.34) | 0.323 | 25.6 | (-33.6, 58.5) |
| Primary | 1.37 | (0.91, 2.08) | 1.33 | (0.81, 2.17) | 0.260 | -32.6 | (-116.7, 18.8) |
| Secondary / College | 1.17 | (0.73, 1.86) | 1.07 | (0.63, 1.82) | 0.799 | -7.1 | (-81.8, 36.9) |

The reference category for each variable is indicated with a dash (-). For the categorical variables Age, Socio-economic Status (SES) and Education, the Wald Test P-values are provided in the rows for the specific categories, and the P-value from the global Likelihood Ratio Test for the overall association of the variable is also provided, marked with an asterisk (*), in the heading row.

**Table K. Nigeria 2016 - Results of crude and multivariable conditional logistic regression**

|  | **Crude OR** | **95% CI** | **Adjusted OR** | **95% CI** | **P-value** | **PE %** | **95% CI** |
| --- | --- | --- | --- | --- | --- | --- | --- |
| **SMC** |  |  |  |  |  |  |  |
| within past 28 days | 0.218 | (0.132, 0.360) | 0.169 | (0.092, 0.309) | <0.001 | 83.1 | (69.1, 90.8) |
| 29-42 days ago | 0.41 | (0.20, 0.85) | 0.36 | (0.15, 0.87) | 0.024 | 63.7 | (12.7, 84.9) |
| 43+ days ago | - | - | - | - | - | - | - |
|  |  |  |  |  |  |  |  |
| **Sex** |  |  |  |  |  |  |  |
| Female | - | - | - | - | - | - | - |
| Male | 0.77 | (0.60, 0.98) | 0.76 | (0.56, 1.04) | 0.081 | 24.2 | (-3.5, 44.5) |
|  |  |  |  |  |  |  |  |
| **Age** |  |  |  |  | **<0.001*** |  |  |
| under 1 year | 0.26 | (0.16, 0.42) | 0.28 | (0.15, 0.53) | <0.001 | 71.9 | (47.3, 85.1) |
| 1 year | 0.56 | (0.37, 0.85) | 0.63 | (0.37, 1.08) | 0.093 | 36.8 | (-8.0, 63.0) |
| 2 years | 0.61 | (0.41, 0.92) | 0.59 | (0.35, 0.99) | 0.044 | 41.5 | (1.5, 65.3) |
| 3 years | 0.65 | (0.43, 0.97) | 0.79 | (0.47, 1.34) | 0.385 | 20.8 | (-34.0, 53.2) |
| 4 years | - | - | - | - | - | - | - |
|  |  |  |  |  |  |  |  |
| **LLIN** | - | - | - | - | - | - | - |
| no |  |  |  |  |  |  |  |
| yes | 0.24 | (0.16, 0.35) | 0.25 | (0.16, 0.38) | <0.001 | 75.1 | (62.2, 83.6) |
|  |  |  |  |  |  |  |  |
| **SES** |  |  |  |  | **0.042*** |  |  |
| Highest | - | - | - | - | - | - | - |
| High | 0.98 | (0.59, 1.62) | 0.92 | (0.50, 1.70) | 0.801 | 7.6 | (-70.4, 49.9) |
| Medium | 0.55 | (0.31, 0.98) | 0.57 | (0.29, 1.13) | 0.105 | 43.0 | (-12.5, 71.1) |
| Low | 0.85 | (0.50, 1.44) | 0.68 | (0.35, 1.29) | 0.236 | 32.5 | (-29.3, 64.7) |
| Lowest | 2.17 | (1.23, 3.84) | 1.38 | (0.67, 2.84) | 0.377 | -38.3 | (-184.4, 32.7) |
|  |  |  |  |  |  |  |  |
| **Education** |  |  |  |  | **0.315*** |  |  |
| None / Koranic | - | - | - | - | - | - | - |
| Primary | 0.51 | (0.35, 0.75) | 0.76 | (0.47, 1.23) | 0.263 | 24.0 | (-22.9, 53.0) |
| Secondary/College | 0.65 | (0.38, 1.09) | 1.06 | (0.53, 2.13) | 0.864 | -6.2 | (-112.5, 46.9) |

The reference category for each variable is indicated with a dash (-). For the categorical variables Age, Socio-economic Status (SES) and Education, the Wald Test P-values are provided in the rows for the specific categories, and the P-value from the global Likelihood Ratio Test for the overall association of the variable is also provided, marked with an asterisk (*), in the heading row.

**Table L. The Gambia 2016 - Results of crude and multivariable conditional logistic regression**

|  | **Crude OR** | **95% CI** | **Adjusted OR** | **95% CI** | **P-value** | **PE %** | **95% CI** |
| --- | --- | --- | --- | --- | --- | --- | --- |
| **SMC** |  |  |  |  |  |  |  |
| within past 28 days | 0.081 | (0.041, 0.159) | 0.081 | (0.039, 0.170) | <0.001 | 91.9 | (83.0, 96.1) |
| 29-42 days ago | 0.27 | (0.12, 0.59) | 0.22 | (0.09, 0.53) | 0.001 | 77.9 | (46.6, 90.8) |
| 43+ days ago | - | - | - | - | - | - | - |
|  |  |  |  |  |  |  |  |
| **Sex** |  |  |  |  |  |  |  |
| Female | - | - | - | - | - | - | - |
| Male | 0.82 | (0.60, 1.11) | 0.83 | (0.57, 1.19) | 0.302 | 17.4 | (-18.7, 42.6) |
|  |  |  |  |  |  |  |  |
| **Age** |  |  |  |  | **<0.001*** |  |  |
| under 1 year | 0.18 | (0.08, 0.42) | 0.19 | (0.08, 0.48) | <0.001 | 81.0 | (52.0, 92.5) |
| 1 year | 0.51 | (0.31, 0.84) | 0.49 | (0.27, 0.86) | 0.013 | 51.5 | (14.1, 72.7) |
| 2 years | 0.85 | (0.55, 1.31) | 0.79 | (0.47, 1.33) | 0.374 | 20.9 | (-32.6, 52.8) |
| 3 years | 1.02 | (0.66, 1.56) | 1.14 | (0.69, 1.89) | 0.613 | -13.9 | (-88.7, 31.2) |
| 4 years | - | - | - | - | - | - | - |
|  |  |  |  |  |  |  |  |
| **LLIN** |  |  |  |  |  |  |  |
| no | - | - | - | - | - | - | - |
| yes | 0.92 | (0.57, 1.47) | 1.29 | (0.71, 2.34) | 0.398 | -29.1 | (-133.6, 28.6) |
|  |  |  |  |  |  |  |  |
| **SES** |  |  |  |  | **<0.001*** |  |  |
| Highest | - | - | - | - | - | - | - |
| High | 0.53 | (0.33, 0.85) | 0.56 | (0.33, 0.93) | 0.025 | 44.3 | (7.2, 66.6) |
| Medium | 0.41 | (0.24, 0.67) | 0.34 | (0.19, 0.62) | <0.001 | 65.6 | (38.3, 80.8) |
| Low | 0.19 | (0.11, 0.36) | 0.22 | (0.11, 0.44) | <0.001 | 77.6 | (55.6, 88.7) |
| Lowest | 0.30 | (0.17, 0.53) | 0.28 | (0.14, 0.55) | <0.001 | 72.1 | (45.4, 85.7) |
|  |  |  |  |  |  |  |  |
| **Education** |  |  |  |  | **0.222*** |  |  |
| None / Koranic | - | - | - | - | - | - | - |
| Primary or higher | 0.82 | (0.53, 1.25) | 0.74 | (0.45, 1.23) | 0.250 | 25.8 | (-23.3, 55.3) |

The reference category for each variable is indicated with a dash (-). For the categorical variables Age, Socio-economic Status (SES) and Education, the Wald Test P-values are provided in the rows for the specific categories, and the P-value from the global Likelihood Ratio Test for the overall association of the variable is also provided, marked with an asterisk (*), in the heading row.

**Table M. Protective efficacy of long-lasting insecticidal net (LLIN) against malaria from the individual studies**

|  | **Protective efficacy, % (95% CI)** | **P-value** |
| --- | --- | --- |
| Mali-2015 | 68.2 (-226.6, 96.9) | 0.335 |
| The Gambia-2015 | 49.9 (19.1, 69.0) | 0.005 |
|  |  |  |
| Burkina-2016 | 47.4 (-4.4, 73.5) | 0.066 |
| Chad-2016 | -33.0 (-122.1, 20.3) | 0.275 |
| Mali-2016 | -27.6 (-220.4, 49.2) | 0.605 |
| Nigeria-2016 | 75.1 (62.2, 83.6) | <0.001 |
| The Gambia-2016 | -29.1 (-133.6, 28.6) | 0.398 |

Results are taken from the tables above (supplementary tables S3-S9), presented here for ease of reference. Protective efficacy calculated as 1-OR, expressed as a percentage, from the multivariable model, i.e. adjusted for recent SMC, age, sex and education, except for Gambia, 2015, adjusted for recent SMC, age and education (i.e. not adjusted for sex).

The high protective efficacy for LLIN in Nigeria 2016 may partly be explained by association of LLIN use with SES (LLIN use was more frequent among children living in households with higher SES, whereas the reverse was true for SMC).
